# High-frequency stimulation of the subthalamic nucleus modifies the expression of vesicular glutamate transporters in basal ganglia in a rat model of Parkinson’s disease

**DOI:** 10.1186/1471-2202-14-152

**Published:** 2013-12-05

**Authors:** Mathieu Favier, Carole Carcenac, Guillaume Drui, Sabrina Boulet, Salah El Mestikawy, Marc Savasta

**Affiliations:** 1 Institut National de la Santé et de la Recherche Médicale, Unité 836, Grenoble Institut des Neurosciences, Equipe Dynamique et Physiopathologie des Ganglions de la Base, Grenoble F-38043, Cedex 9, France; 2 Université de Grenoble, Grenoble F- 38042, France; 3 Centre Hospitalier Universitaire de Grenoble, BP217, Grenoble F-38043, France; 4 Institut National de la Santé et de la Recherche Médicale (INSERM), U952, Université Pierre et Marie Curie, Paris F-75005, France; 5 Centre National de la Recherche Scientifique (CNRS) UMR 7224, Paris F-75005, France; 6 Université Pierre et Marie Curie (UPMC) Paris 06, Pathophysiology of Central Nervous System Disorders, Paris F-75005, France; 7 Department of Psychiatry, Douglas Hospital Research Center, McGill University, 6875, boulevard Lasalle, Verdun, QC, Canada

**Keywords:** High frequency stimulation, Subthalamic nucleus, Parkinson’s disease, Basal Ganglia, 6-OHDA-lesion, Rat, Glutamate, Vesicular glutamate transporters

## Abstract

**Background:**

It has been suggested that glutamatergic system hyperactivity may be related to the pathogenesis of Parkinson’s disease (PD). Vesicular glutamate transporters (VGLUT1-3) import glutamate into synaptic vesicles and are key anatomical and functional markers of glutamatergic excitatory transmission. Both VGLUT1 and VGLUT2 have been identified as definitive markers of glutamatergic neurons, but VGLUT 3 is also expressed by non glutamatergic neurons. VGLUT1 and VGLUT2 are thought to be expressed in a complementary manner in the cortex and the thalamus (VL/VM), in glutamatergic neurons involved in different physiological functions. Chronic high-frequency stimulation (HFS) of the subthalamic nucleus (STN) is the neurosurgical therapy of choice for the management of motor deficits in patients with advanced PD. STN-HFS is highly effective, but its mechanisms of action remain unclear. This study examines the effect of STN-HFS on VGLUT1-3 expression in different brain nuclei involved in motor circuits, namely the basal ganglia (BG) network, in normal and 6-hydroxydopamine (6-OHDA) lesioned rats.

**Results:**

Here we report that: 1) Dopamine(DA)-depletion did not affect VGLUT1 and VGLUT3 expression but significantly decreased that of VGLUT2 in almost all BG structures studied; 2) STN-HFS did not change VGLUT1-3 expression in the different brain areas of normal rats while, on the contrary, it systematically induced a significant increase of their expression in DA-depleted rats and 3) STN-HFS reversed the decrease in VGLUT2 expression induced by the DA-depletion.

**Conclusions:**

These results show for the first time a comparative analysis of changes of expression for the three VGLUTs induced by STN-HFS in the BG network of normal and hemiparkinsonian rats. They provide evidence for the involvement of VGLUT2 in the modulation of BG cicuits and in particular that of thalamostriatal and thalamocortical pathways suggesting their key role in its therapeutic effects for alleviating PD motor symptoms.

## Background

It is long recognized that the degeneration of dopaminergic neurons induces an abnormal activation of glutamate systems in the basal ganglia (BG) that is central to the pathophysiology of Parkinson’s disease (PD) [1-4]. Glutamate mediated mechanisms are also thought to play a role in the development of dyskinesias with long-term administration of L-3,4-dihydroxyphenylalanine (L-DOPA), the most efficient treatment for PD. Many experimental studies also evidence that dopamine denervation induces an increase in corticostriatal glutamate [5-11] and that L-DOPA-induced dyskinesia (LID) are linked to BG network glutamate transmission abnormalities [12,13]. Microdialysis studies have suggested that dopamine lesion may also increase glutamate transmission in the BG output structures, substantia nigra pars reticulata (SNr) [5,14-16] and entopeduncular nucleus [6], presumably as a result of the abnormal activation of the subthalamic nucleus (STN) [17].

Three subtypes of vesicular glutamate transporters have been identified: VGLUT1, 2 and 3 [18]. These transporters mediate glutamate uptake inside presynaptic vesicles and are anatomical and functional markers of glutamatergic excitatory transmission [19-25]. VGLUT1-3 are very similar in structure and function, but are used by different neuronal populations. VGLUT1 and VGLUT2 are expressed by the cortical and subcortical neurons respectively. VGLUT3 is expressed by nonglutamatergic neurons, such as cholinergic striatal interneurons, a GABAergic interneuron subpopulation from the cortex and hippocampus and serotoninergic neurons from the dorsal and medial raphe nuclei [22,26].

Since the 1990s, High Frequency Stimulation (HFS) of the STN has become an effective surgical treatment of late-stage Parkinson’s disease (PD), improving all motor symptoms in PD patients, particularly in those who experience motor fluctuations [27-29]. However, the mechanisms underlying the improvement in symptoms remain unclear [30-32]. Beyond its local effect on STN activity, we know that, by activating axons, STN-HFS may generate widespread and heterogeneous distal effects throughout the BG network [32,33]. Indeed, we have already reported in previous studies that in intact or 6-OHDA (6-hydroxydopamine)-lesioned rats, STN-HFS increases extracellular glutamate in the striatum, the globus pallidus and the SNr [14-16,34].

The present study analyzed the effects of DA depletion and for the first time those of STN-HFS on VGLUT1-3 expression in several BG nuclei, by using immunoradioautography with affinity-purified rabbit VGLUT1, VGLUT2 or VGLUT3 antiserum.

We found that DA-depletion did not affect VGLUT1 and VGLUT3 expression in almost all BG structures studied while that of VGLUT2 significantly decreased. Interestingly, STN-HFS did not affect VGLUT1-3 expression in normal rats, but systematically increased their expression in most of the BG nuclei studied in DA-depleted animals.

According to the changes of VGLUT1-3 expression observed and to their known anatomical localization, we suggest that STN-HFS may achieve its therapeutic effect, at least in part, through normalization of the thalamostriatal and thalamocortical pathways.

## Methods

### Animals

Adult (5 to 7 weeks old) male Sprague–Dawley rats (Janvier, Le Genest St Isle, France), weighing 180 to 270 g, were housed in an animal room on a 12-hour light/dark cycle, with food and water supplied *ad libitum*. This study was carried out in strict accordance with the recommendations of the European Community Council Directive of 24 November 1986 (86/609/EEC) concerning the care of laboratory animals, French Ministry of Agriculture regulations (Direction Départementale de la Protection des Populations, Préfecture de l’Isère, France, Grenoble Institute of Neuroscience, agreement number: A 38-516-10-008; Marc Savasta, permit number 38-10-08, Carole Carcenac permit number 38-10-23) and French guidelines for the use of live animals in scientific investigations. The protocol was approved by the Committee on the Ethics of Animal Experiments of the “Grenoble Institute of Neuroscience ethical committee” agreement number 04. All surgery was performed under a mixture of xylazine and ketamaine and all efforts were made to minimize the number of animal used and their suffering. All operated rats were intraperitoneally treated with Rimadyl (1 ml.kg^-1^) to prevent post-surgery suffering.

### Lesion procedure

Forty rats (*n* = 40) were anesthetized with a mixture of xylazine (10 mg.kg^-1^, intraperitoneal) and ketamine (100 mg.kg^-1^, intraperitoneal) and secured in a Kopf stereotaxic apparatus (Phymep, Paris, France). All animals received desipramine (25 mg/kg s.c.) pretreatment, to protect noradrenergic neurons. Lesioned animals (*n* = 20) received a unilateral injection of 9 μg of 6-hydroxydopamine (6-OHDA) (Sigma, St. Quentin-Fallavier, France) dissolved in 3 μl of 0.9% sterile NaCl supplemented with 0.2% ascorbic acid, administered at a flow rate of 0.5 μl · min^-1^ to the left SNc. An identical procedure was used for controls (*n* = 20) but with the injection of NaCl 0.9%. The stereotaxic coordinates for the injection site relative to the bregma were as follows: anteroposterior (AP), -5.3 mm; lateral (L), +2.35 mm; dorsoventral (DV), -7.5 mm, with the incisor bar at 3.3 mm below the interaural plane, according to the stereotaxic atlas of Paxinos and Watson [35]. After injections, animals were kept warm and allowed to recover from the anesthetic before being returned to the animal house for three weeks until the stimulation experiments. This time interval was left to allow the DA system degeneration induced by the neurotoxin to stabilize.

### Implantation of the stimulation electrode

Rats from the two experimental groups (sham-operated controls, *n* = 20, and 6-OHDA lesioned, *n* = 20) were first anesthetized by the inhalation (1 l.min^-1^) of a mixture of 3% isoflurane in air (the air used being composed of 22% O_2_, 78% N_2_) and mounted in a stereotaxic frame (David Kopf Instruments, Tujunga, CA). The dorsal skull was exposed and holes were drilled for the implantation of the stimulation electrode into the left STN. During the implantation and stimulation procedure, anesthesia was maintained with an inhaled mixture of 1% isoflurane in air (1 l.min^-1^) and body temperature was maintained at 37°C with a feedback-controlled heating pad (Harvard Apparatus, Edenbridge, UK). Stereotaxic coordinates were chosen according to the atlas of Paxinos and Watson [35] and were as follows relative to the bregma: AP, -3.7 mm; L, +2.4 mm; and DV, -7.8 mm as previously described [14-16,34,36].

### Electrical stimulation

For electrical stimulation, we used a concentric stimulating bipolar electrode (SNEX 100, Rhodes Medical Instruments, Woodland Hills, CA), with an outer diameter of 250 μm and a distance between the poles of 1 mm. Stimuli were delivered under anesthesia during 4 hours with a World Precision Instrument (Stevenage, UK) acupulser and stimulus isolation units giving a rectangular pulse. This duration of stimulation (> 1 h) was chosen to be sure that the proteic expression of VGLUTs can be detected and stabilized and almost corresponds to that used in previous studies analyzing mRNA levels of different target proteins of basal ganglia circuits [37]. As previously reported, the stimulation parameters (130 Hz, 60 μs, 200 μA) matched those routinely used in Parkinsonian patients [14,34,36]. At the end of each experiment, an electrical lesion was created in the STN so that the position of the electrode could be checked post-mortem. In control rats (sham-operated and 6-OHDA-lesioned) the stimulation was never switched “on”.

### Histology

At the end of the electrical stimulation, all animals were perfused transcardially with 0.9% saline, under chloral hydrate anesthesia. Brains were rapidly removed and frozen in cooled (−40°C) isopentane, then stored at −20°C. Serial frontal sections (14-μm thick) were cut with a cryostat (Microm HM 500, Microm, Francheville, France), collected on microscopic slides and stored at −20°C. Tissue sections from different BG nuclei and related structures (striatum (caudate-putamen), nucleus accumbens, motor and somatosensory cortices, thalamus (VL/VM), subthalamic nucleus, globus pallidus and substantia nigra pars reticulata (SNr)) were selected to analyze changes in VGLUT expression.

The correct location of the stimulation electrode was checked by collecting several subthalamic tissue sections (n = 12 sections per stimulated rat) (14 μm thick from AP, -3,6 to −4,3 mm relative to the bregma, Paxinos and Watson, [35]) and counterstaining with cresyl violet. The tip of the electrode was systematically implanted directly in the STN at the top of its dorsal part. These histological controls were systematically carried out for all the animals in each experimental group. All animals with incorrectly positioned stimulation electrodes were excluded (controls, *n* = 3 and 6-OHDA lesioned, *n* = 4).

### TH-immunohistochemistry

We assessed the extent of the dopaminergic denervation induced by nigral 6-OHDA injection by TH immunostaining on striatal and nigral sections from the fixed brains of lesioned animals. TH immunostaining was carried out as previously described [14]. Briefly, striatal and nigral tissue sections from 6-OHDA-lesioned rats were mounted on silane-coated microscope slides. Tissue sections were postfixed in 4% paraformaldehyde, thoroughly washed with Tris buffered-saline (TBS, 0.1 M, pH 7.4) and incubated for 1 hour in 0.3% Triton X-100 in TBS (TBST) and 3% normal goat serum (NGS, Sigma-Aldrich, St Quentin Fallavier, France). They were then incubated with primary antisera diluted in TBST supplemented with 1% normal goat serum (NGS) for 24 h, at 4°C. The antiserum was diluted 1:500 for TH staining (mouse monoclonal antibody; Chemicon, Temecula, CA). Antibody binding was detected with avidin-biotin-peroxidase conjugate (Vectastain ABC Elite, Vector Laboratories, Burlingame, CA), with 3, 3’-diaminobenzidine as the chromagen. The detection reaction was allowed to proceed for one to three minutes, as previously described. Sections were dehydrated in a series of graded ethanol solutions, cleared in xylene, mounted in DPX (DBH Laboratories Supplies, Poole, UK) and covered with a coverslip for microscopy.

### VGLUT 1–3 immunoradioautography

Tissue sections were air-dried, post-fixed by immersion in fixative (4% PFA), and then washed in PBS. Nonspecific binding sites were saturated by incubation with 3% bovine serum albumin (BSA) in PBS, 1% NGS and 2 mM NaI (buffer A). Sections were incubated overnight at 4°C in buffer A supplemented with affinity-purified rabbit VGLUT1, VGLUT2 or VGLUT3 antiserum (dilution 1/10000 for VGLUT1 and VGLUT2, 1/5000 for VGLUT3, from Dr Salah El Mestikawy), and then for 1 hour with an affinity-purified goat anti-rabbit [^125^I] IgG (0.25 μCi/ml, Perkin Elmer, Paris, France) in buffer A supplemented with 0.02% sodium azide. The sections were rinsed in water, dried and placed against X-ray films (Biomax MR, Kodak) for 9 to 11 days.

The specificity of all antisera used in this study have been previously validated by our group (Gras et al. [22], [38]; Herzog et al. [23], [26]). For each labeled section, a background value was estimated by measuring optical density in the corpus callosum, since this structure is devoided of specific staining for VGLUT 1–3 antibodies. This background value was then systematically subtracted from the optical density values obtained for each corresponding section.

### Quantification and statistical analysis

For the evaluation of the extent of DA-denervation, striatal and nigral TH immunostained sections were directly processed by using the Calopix software of the computerized image analysis system (TRIBVN, 2.9.2 version, Châtillon, France). Six TH-immunostained sections from each structure (striatum and SNc) and for each rat were used for quantification. The loss of TH immunostaining in the SNc or in the striatum was evaluated by comparing the total surface of both structures, as revealed by the TH immunolabelling, in normal and lesioned animals.

For quantification of VGLUT1-3 contents, four AP levels (+1, -0.92, -3.8 and −5.5 mm relative to bregma (Paxinos et Watson, [35])) were choosen. For each rat, three stained sections of the same AP level were used for quantification and the triplicate OD values obtained for each structure analyzed were averaged. Immunoradioautograms obtained from X-ray films were analyzed with Autoradio V4.03 software (SAMBA Technologies, Meylan, France). Values of optical densities measured from each structure analyzed are expressed as a mean ± standard error (SEM) in Table 1. Histograms presented in figures show the mean ± standard error of the mean (SEM) of optical densities expressed as a percentage of control values. Data were analyzed for each brain structure by Kruskal-Wallis tests with SigmaStat 3.1 software. Post-hoc analyses were carried out with the Dunn’s method.

**Table 1:** Effect of 6-OHDA-lesion and STN-HFS on bilateral changes of optical density measurements of immunoreactive signals for VGLUT1-3

	**VGLUT1**				**VGLUT2**				**VGLUT3**			
	**Ipsilateral side**		**Controlateral side**		**Ipsilateral side**		**Controlateral side**		**Ipsilateral side**		**Controlateral side**	
**A**	**Controls**	**6-OHDA**	**Controls**	**6-OHDA**	**Controls**	**6-OHDA**	**Controls**	**6-OHDA**	**Controls**	**6-OHDA**	**Controls**	**6-OHDA**
CPu	51,35 (±3,2)	53,33 (±1,9)	48,78 (±3,4)	52,59(±1,7)	**20,15** (±2,38)	12,64*(±1,5) **-37%**	**19,76** (±2,3)	13,38*(±0,6) **-33%**	16, 65(±0,8)	18,15 (±1,7)	15,37 (±0,8)	16, 33 (±3,1)
PM Cx	55,54 (±3,3)	48,3 (±2,1)	55,81 (±2,9)	48,07 (±2,8)	**18,56** (±1,7)	10,93*(±0,6) **-42%**	**18,5** (±2,1)	11,12*(±0,6) **-40%**	14,34 (±0,9)	13,09 (±2,4)	14,39 (±0,9)	13, 02 (±2,5)
SS Cx	45,93 (±3)	35,37 (±1,6)	45,93 (±1,6)	38,93 (±1,6)	**15,79** (±1,81)	9,22*(±0,4) -**42%**	**16,44** (±1,8)	10,57*(±0,6) **-36%**	12,01 (±0,6)	10,28 (±2,1)	12,89 (±0,7)	11,63 (±2,4)
Acb	54,15 (±2,8)	63,43 (±2,8)	51,52 (±3,6)	59,65 (±3,1)	**22,55** (±2,7)	13,56*(±2,1) **-40%**	**22,14** (±2,6)	13,91*(±2,1) **-47%**	18,84 (±0,9)	23,33 (±1,1)	19,04 (±1,1)	23 (±1,2)
Thalamus	41,18 (±3,4)	34,15 (±1,7)	41,86 (±3,3)	32,47 (±1,1)	**16,83** (±2,3)	8,85*(±0,4) **-48%**	**17,49** (±2,4)	9,05*(±0,7) -**48%**	11,38 (±0,5)	9,85 (±1,3)	11,43 (±0,5)	9,79 (±1,1)
STN	21,13 (±2,7)	23, 56 (±4,6)	19,25 (±2,5)	20,65 (±4,2)	**16,83** (±2,1)	7,66*(±1,3) **-55%**	**15,53** (±2,3)	9,32*(±0,5) **-40%**	ND	ND	ND	ND
GP	ND	ND	ND	ND	12,78 (±1,9)	7,97(±1)	13,39 (±1,7)	8,83 (±1,1)	ND	ND	ND	ND
SNr	ND	ND	ND	ND	51,1 (±2,4)	11,26*(±0,3) **-25%**	**15,76** (±2,2)	11,39*(±0,5) **-28%**	ND	ND	ND	ND
	**Ipsilateral side**		**Controlateral side**		**Ipsilateral side**		**Controlateral side**		**Ipsilateral side**		**Controlateral side**	
**B**	**6-OHDA**	**6-OHDA + STN-HFS**	**6-OHDA**	**6-OHDA + STN-HFS**	**6-OHDA**	**6-OHDA + STN-HFS**	**6-OHDA**	**6-OHDA + STN-HFS**	**6-OHDA**	**6-OHDA + STN-HFS**	**6-OHDA**	**6-OHDA + STN-HFS**
CPu	**53,33** (±1,8)	65,07*(±3,4) **+22%**	52,59 (±1,7)	64,43(±3,2) +23%	**12,64** (±1,5)	17,81*(±1,4) +41%	**13,38** (±0,6)	17,61*(±1,8) +32%	**18,15** (±1,7)	26,13*(±2,9) **+44%**	**16,33** (±3,1)	24,88*(±1,8) **+53%**
PM Cx	**48,3** (±2,2)	63,08*(±3,3) **+31%**	**48,07** (±2,8)	64, 79*(±3,5) **+35%**	**10,93** (±0,6)	16,93*(±1,6) **+55%**	**11,12** (±0,6)	16,86*(±1,8) **+52%**	**13,09** (±2,4)	20*(±1,9) **+53%**	**13,02** (±2,5)	16,62*(±1,7) **+51%**
SS Cx	**35,75** (±1)	54,25*(±3,8) **+52%**	**38,93** (±1,6)	55,08*(±3,8) **+41%**	**9,22** (±0,4)	14,03*(±1,4) +52%	**10,57** (±0,6)	13,38*(±1,6) **+27%**	**10,28** (±2,1)	20,44*(±2,1) **+99%**	**11,63** (±2,4)	16,78*(±1,4) **+44%**
Acb	63,43 (±2,8)	65,39 (±3,9)	59,65 (±4,1)	65,06 (±4,1)	**13,56** (±2,1)	20,25*(±1,3) **+49%**	**13,91** (±2,1)	18,78*(±1,2) **+35%**	**23,23** (±1,1)	31,7* (1,2) **+36%**	**23** (±1,2)	31,89*(±1,2) **+38%**
Thalamus	**34,15 (±1,7)**	48,1*(±3,8) **+41%**	**32,47(±1,1)**	**46,37** (±3,6) **+43%**	**8,85** (±0,4)	14,63*(±1,6) **+65%**	**9,05** (±0,7)	13,5*(±1,5) **+449%**	**9,85** (±1,3)	20,3*(±1,6) **+106%**	**9,79** (±1,1)	19,38*(±2,5) **+98%**
STN	23,56 (±4,6)	17,78 (±2,6)	20,65 (±4,2)	19,34 (±3)	**7,66** (±1,3)	14,79*(±1,5) **+93%**	**9,32** (±0,5)	15,94*(±1,9) **+71%**	ND	ND	ND	ND
GP	ND	ND	ND	ND	7.97 (±1)	10,18 (±0,9)	8,83 (±1,1)	9,61 (±1)	ND	ND	ND	ND
SNr	ND	ND	ND	ND	**11,26** (±0,3)	15,84*(±1,3) **+41%**	**11,39** (±0,5)	16,64*(±1,4) **+46%**	ND	ND	ND	ND

**A,** Modifications of VGLUT1-3 expression induced by unilateral 6-OHDA-lesion of SNc (control rats, n = 9; 6-OHDA rats, n = 6). *p<0.05, vs control group.

**B,** Modifications of VGLUT1-3 expression induced by unilateral STN-HFS in 6-OHDA lesioned rats (6-OHDA rats, n = 6; 6-OHDA rats + STN-HFS, n = 10). *p<0.05, vs non stimulated 6-OHDA-lesioned group. Values of optical densities measurements are expressed as a mean ± standard error (SEM). Data were analyzed for each brain structure by Kruskal-Wallis tests with SigmaStat 3.1 software. Post-hoc analyses were carried out with the Dunn’s method. Bold numbers correspond to significant differences. Ipsilateral side: related to the lesion and/or stimulation side; Controlateral side: related to the lesion and/or stimulation side; Acb, Accumbens nucleus; CPu, Caudate Putamen (striatum); GP, Globus Pallidus; PM Cx, Premotor Cortex; SNr, Substantia nigra *pars reticulata*; SS Cx, Somatosensory Cortex; STN, Subthalamic nucleus; Thalamus (VL/VM).

## Results and discussion

### Histological controls of the extent of the dopamine lesion and of electrode location

**Figure 1:**
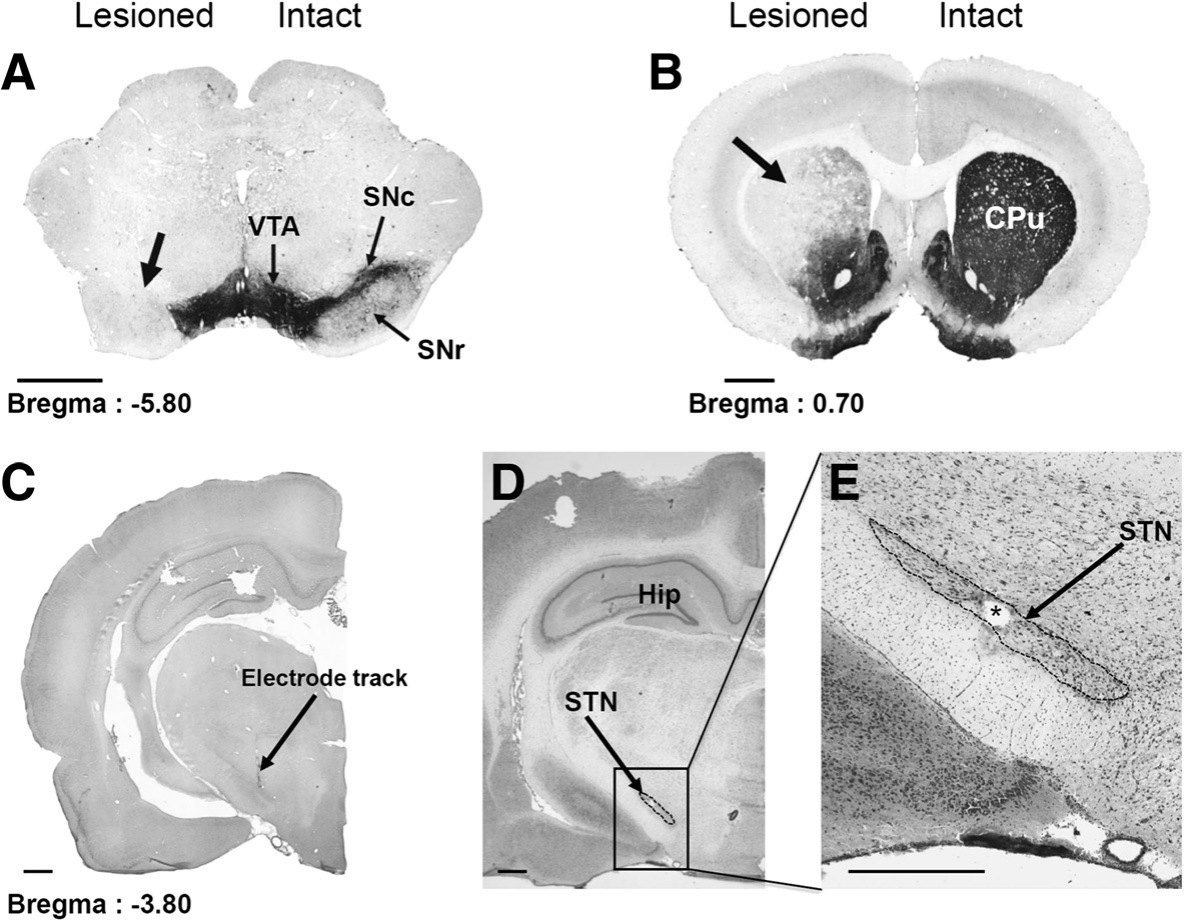
**Photographs of TH-immunostained coronal rat-brain sections at the nigral (A) and striatal (B) levels and of cresyl violet-stained coronal rat-brain sections at subthalamic (C, D and E) levels in 6-OHDA-lesioned rats.** Note, on the lesioned side (left), the loss of dopaminergic cells in the SNc **(A)** and the loss of dopaminergic terminals in the striatum **(B)**. Note also the correct implantation of the stimulation electrode within the STN **(C, D, E). C**, The arrow indicates the electrode track. **E**, The asterisk indicates the point of stimulation. CPu: Caudate Putamen; Hip, Hippocampus; SNc, Substantia nigra pars compacta; SNr, Substantia nigra *pars reticulata*; STN, Subthalamic nucleus; VTA, Ventral Tegmental Area. Scale bar, 0.75 mm.

Three weeks after the unilateral injection of 6-OHDA, all lesioned animals presented a substantial loss of TH immunostaining in the ipsilateral SNc and the striatum (caudate-putamen nucleus), as shown by comparison with the contralateral side (Figure 1A, B) or with control animals. An analysis of densitometric measurements of TH immunostaining showed an absence of statistical difference between the two lesioned groups (non stimulated and stimulated).

In DA-depleted animals, the loss of SNc TH + neurons was evaluated by comparing the total SNc surface on the intact side with the homologous area on the lesioned side. A loss of 92 ± 5% (p < 0.001) of TH immunolabeled surface was measured. In the striatum of the same rats, the loss of DA nerve terminals, as revealed by TH immunostaining mainly affected the dorsal part of the striatum (Figure 1B). This loss affected around 83 ± 4% of the striatal surface as compared to the total striatal surface of the control side. In this denervated striatal area, TH immunolabeling, as evaluated by a mean of densitometric values, was decreased by 85 ± 5% (p < 0.001) when compared to the controlateral intact side.

The correct implantation of the stimulation electrode in the STN is illustrated in Figure 1C-E. Figure 1E shows, at a higher magnification, the small electrical lesion (asterisk) created at the end of the experiment, indicating the point stimulated.

### Regional distribution of VGLUT1-3 in control rats (without lesioning and stimulation)

VGLUT1-3 expression was qualitatively analyzed in control rats that had been neither lesioned nor stimulated, to ensure the validity and specificity of the immunoradioautographical staining. Immunoradioautograms from the different sections showed a distribution of VGLUT1-3 similar to that previously reported [26,39], confirming the validity of our VGLUT1-3 staining procedure and the lack of cross-reactivity between the antibodies used.

**Figure 2:**
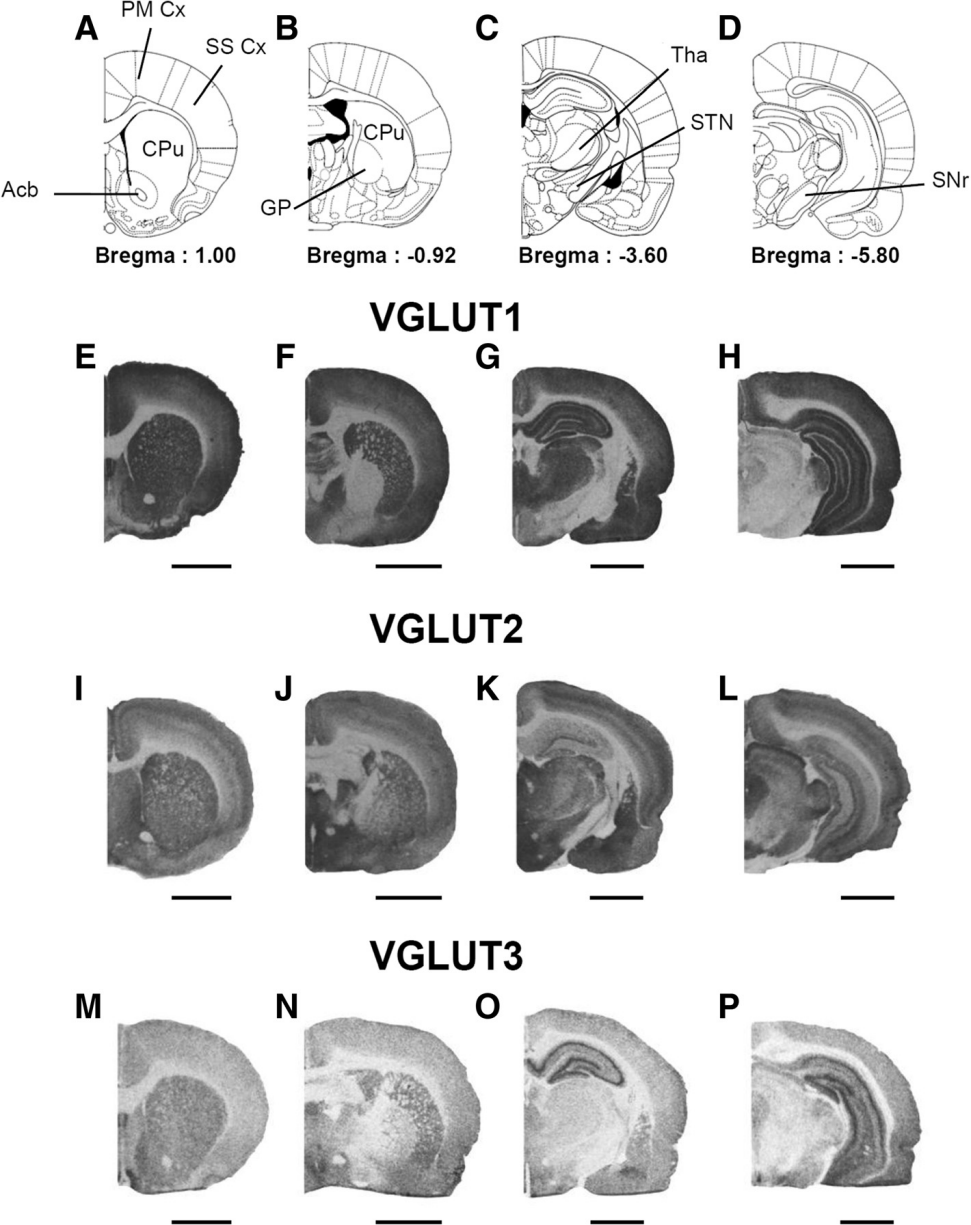
**Regional distribution of VGLUT1-3 proteins in control (without lesion and stimulation) rats. A-D**, Schematic diagrams adapted from the stereotaxic atlas of Paxinos and Watson [1982]. **E-P**, Photographs of immunoradioautograms obtained by incubating coronal rat-brain sections of control rats (non lesioned and non stimulated) with affinity-purified anti-VGLUT1 **(E-H)**, anti-VGLUT2 **(I-L)** and anti-VGLUT3 **(M-P)** antisera and then with anti-rabbit [125I] IgG. Note the different distributions of the three VGLUTs in the brain structures studied. Acb, Accumbens nucleus; Amy, Amygdaloid nucleus; CPu, Caudate Putamen; Cg, Cingulate cortex; GP, Globus Pallidus; Hip, Hippocampus; PM Cx, Premotor Cortex; SNr, Substantia nigra *pars reticulata*; SS Cx, Somatosensory Cortex; STN, Subthalamic nucleus; Tha, Thalamus (VL/VM). Scale bar, 0.4 mm.

VGLUT1 immunostaining was dense in almost all the structures studied, including, especially, the striatum, nucleus accumbens, cortex, the motor part of the thalamus (VL/VM) and hippocampus. By contrast, no VGLUT1 labeling was found in the globus pallidus, the substantia nigra and in most of the brainstem (Figure 2 E-H).

VGLUT2 proteins were detected in almost the same set of structures as VGLUT1 although the density of VGLUT2 immunostaining was slightly lower than that for VGLUT1 in striatal, cortical and thalamic areas, whereas the opposite was observed in many sub-cortical structures. These data are consistent with the well-described complementary pattern of expression of VGLUT1 and VGLUT2 in the rat brain. VGLUT2 staining, unlike that for VGLUT1, was detectable in the substantia nigra *pars reticulata*, hypothalamic nuclei and midbrain, which displayed widespread staining. Different, complementary patterns of immunostaining for VGLUT1 and VGLUT2 were observed in the hippocampus. The density of VGLUT2 proteins was highest in layers IV and VI of the cortex and in the hypothalamus, the central gray matter and the superior colliculus (Figure 2 I-L).

VGLUT3 staining was weaker than that for VGLUT1 and VGLUT2, but was also observed in many different areas. VGLUT3 levels were moderate in the striatum, but high in the hippocampus, with a complementary distribution for VGLUT1 and VGLUT2 (Figure 2M-P).

### Effects of 6-OHDA-lesion and/or STN-HFS on VGLUT1-3 expression

**Figure 3:**
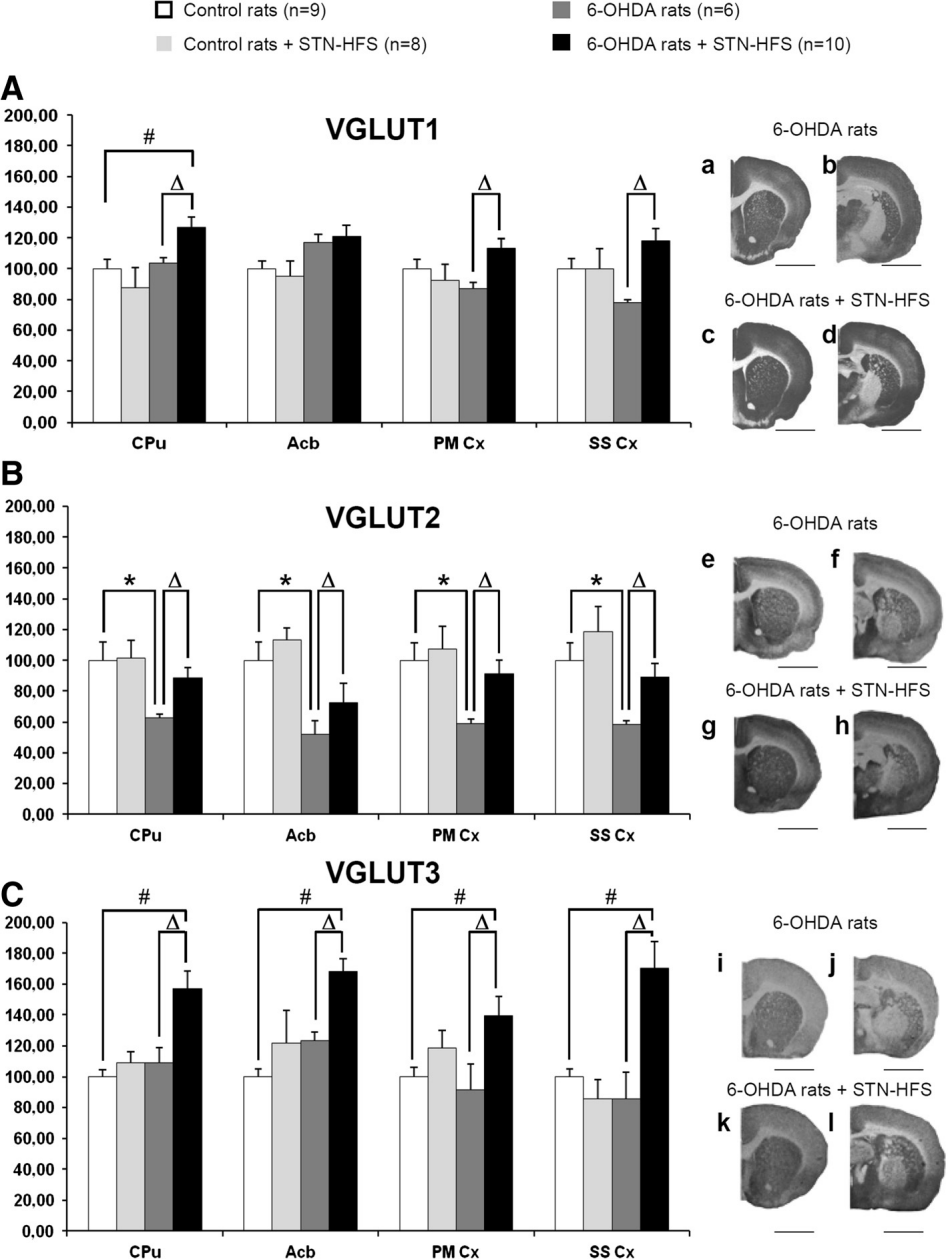
**Effects of 6-OHDA-lesion and STN-HFS on striatal and cortical VGLUT1-3 expression. A-C**, Histograms show the mean ± standard error of the mean (SEM) of optical density values expressed as a percentage of values of control rats (non lesioned and non stimulated). Data were analyzed for each brain structure by Kruskal-Wallis tests with SigmaStat 3.1 software. Post-hoc analyses were carried out with the Dunn’s method. Kruskal-Wallis tests (VGLUT1, CPu, p = 0.024; Acb, p = 0.05; PMCx, p = 0.049; SSCx, p = 0.02), (VGLUT2, CPu, p = 0.009; Acb, p = 0.002; PMCx, p = 0.012; SSCx, p = 0.002), (VGLUT3, CPu, p = 0.003; Acb, p<0.001; PMCx, p = 0.043; SSCx, p = 0.002). a-l, Photographs of immunoradioautograms obtained by incubating coronal rat-brain sections with affinity-purified anti-VGLUT1 (a-d), anti-VGLUT2 (e-h) and anti-VGLUT3 (i-l) antisera and then with 125I-labeled anti-rabbit IgG. Acb, Accumbens nucleus; CPu, Caudate putamen; PM Cx, Premotor cortex; SS Cx, Somatosensory Cortex. *, controls vs 6-OHDA rats; #, controls vs 6-OHDA + STN-HFS rats; Δ, 6-OHDA rats vs 6-OHDA + STN-HFS rats: p < 0.05. Scale bar, 0.4 mm.

**Figure 4:**
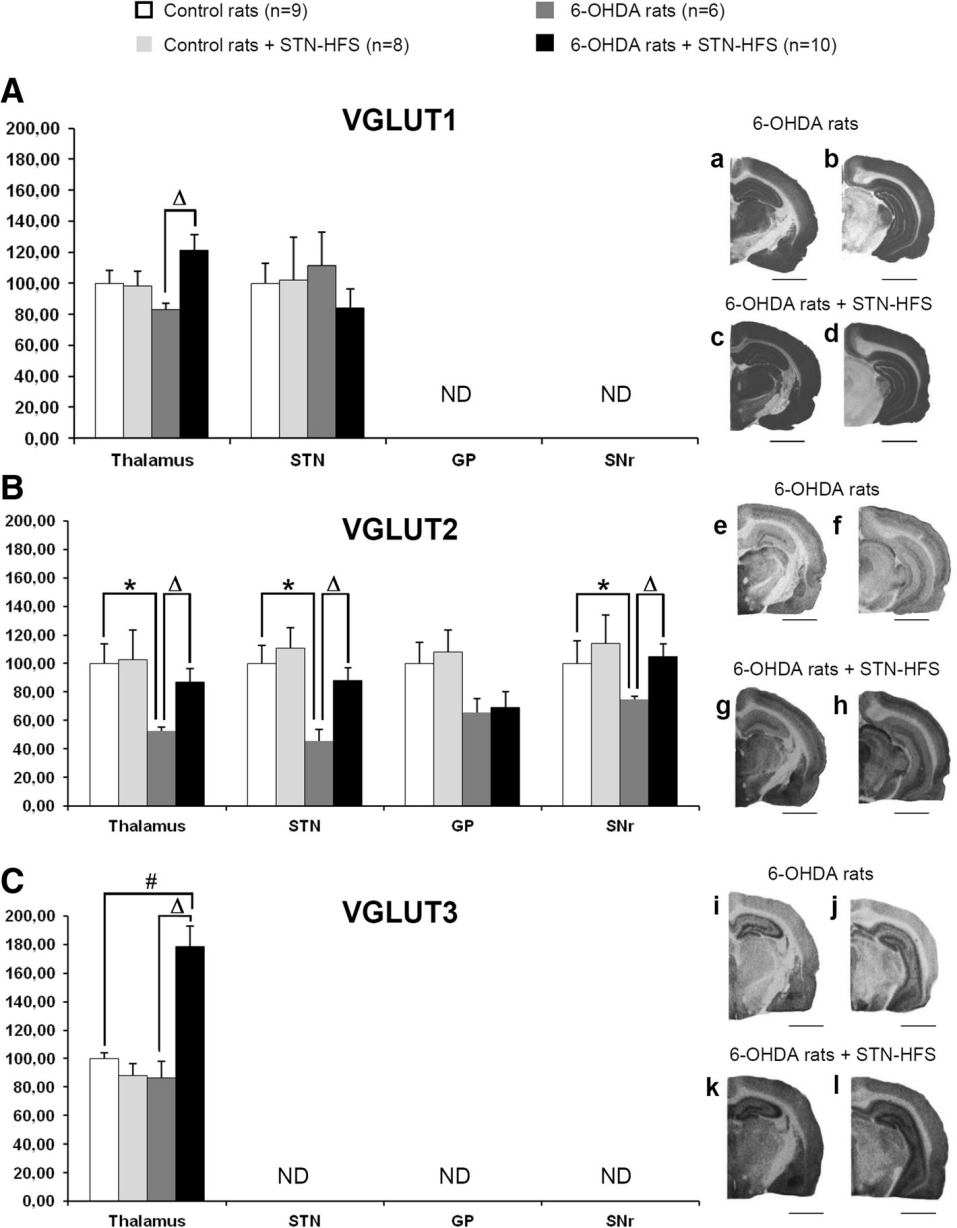
**Effects of 6-OHDA-lesion and STN-HFS on thalamic, pallidal and nigral VGLUT1-3 expression. A-C**, Histograms show the mean ± standard error of the mean (SEM) of optical density values expressed as a percentage of values of control rats (non lesioned and non stimulated). Data were analyzed for each brain structure by Kruskal-Wallis tests with SigmaStat 3.1 software. Post-hoc analyses were carried out with the Dunn’s method. Kruskal-Wallis tests: (VGLUT1, Thalamus VL/VM, p = 0.036; STN, p = 0.536), (VGLUT2, Thalamus (VL/VM); p = 0.036; STN, p = 0.015; SNr, p = 0.049; GP, p = 0.048), (VGLUT3, Thalamus (VL/VM), p<0.001. a-l, Photographs of immunoradioautograms obtained by incubating coronal rat-brain sections with affinity-purified anti-VGLUT1 (a-d), anti-VGLUT2 (e-h) and anti-VGLUT3 (i-l) antisera and then 125I-labeled anti-rabbit IgG. GP, Globus Pallidus; SNr, Substantia nigra pars reticulata; STN, Subthalamic nucleus; Tha, Thalamus (VL/VM). *, controls vs 6-OHDA rats; #, controls vs 6-OHDA + STN-HFS rats; Δ, 6-OHDA rats vs 6-OHDA + STN-HFS rats: p < 0.05. Scale bar, 0.4 mm.

DA lesion and STN stimulation were unilaterally performed in this study. However, we found similar changes of VGLUTs expression on both sides, as revealed by the optical density measurements of immunoreactive signals for VGLUT1-3 in all structures examined (see Table 1). In order to simplify the presentation of our data, we decided to only show the results obtained from the ipsilateral side (the lesioned and/or stimulated side) on Figures 3 and 4. As precised in materials and methods, changes in VGLUTs expression induced by 6-OHDA lesion and STN stimulation were first analyzed by Kruskal-Wallis tests with SigmaStat 3.1 software. Results of these tests for each brain structure are presented in legends of Figures 3 and 4. Post-hoc analyses were then carried out with the Dunn’s method.

#### Effect of the 6-OHDA-lesion on VGLUT1-3 expression

DA depletion did not affect VGLUT1 and VGLUT3 expression whatever the brain structure analyzed. Slight changes were observed in the striatum, nucleus accumbens, somatosensory cortex and thalamus (VL/VM), but they were not statistically significant (Table 1, Figures 3 and 4, white versus dark grey histograms).

By contrast, 6-OHDA lesioning induced a significant decrease in VGLUT2 expression of nearly 50% with respect to that in sham-operated rats (Figures 3 and 4). This decrease was particularly strong in the STN, thalamus (VL/VM) (Figure 4B), and cortical areas (Figure 3B) which displayed decreases of 55%, 48% and 42% with respect to control (non-lesioned) rats, respectively (*p* < 0.05, *n* = 6).

#### Effect of STN-HFS on VGLUT1-3 expression in sham-operated control (non-lesioned) rats and in 6-OHDA-lesioned animals

No significant change in VGLUT1-3 expression was detected after four hours of STN-HFS in sham-operated control rats (non-lesioned) rats. Levels of VGLUT1-3 expression were similar between the two experimental groups (non-lesioned rats with and without STN stimulation) in all different structures studied, as show in Figures 3 and 4.

On the contrary, STN-HFS induced a marked increase in VGLUT1-3 expression in 6-OHDA-lesioned rats. This increase affected almost all the brain structures studied (Table 1, Figures 3 and 4). VGLUT1 levels were always higher for all brain areas studied in stimulated 6-OHDA rats when compared to non-stimulated 6-OHDA-rats except for the nucleus accumbens (Acb) and STN (Figure 3A, a-d, for examples of autoradiographs and Figure 4A, a-d). The largest differences were found in the somatosensory cortex (+52%) and the motor part of the thalamus (VL/VM) (+41%) (*p* < 0.05, *n* = 10). For many brain structures analyzed, VGLUT1 levels measured in stimulated 6-OHDA-rats were comparable to those detected in control (without lesion and stimulation) rats. Interestingly, for the striatum and the thalamus (VL/VM), VGLUT1 levels remained moderately overexpressed versus controls (+27%, *p* < 0.05 and +17%, *p* < 0.05, respectively, *n* = 10) (Figures 3A, a-d, and 4A, a-d).

STN-HFS induced a significant increase in VGLUT2 expression in all structures studied in 6-OHDA-lesioned rats when compared to non-stimulated 6-OHDA-rats. However this increase did not affect the globus pallidus. (Figures 3B, e-h and 4B, e-h). Thus, STN-HFS more or less completely reversed the decrease in VGLUT2 expression induced by the DA-depletion in all structures analyzed.

STN-HFS induced a strong upregulation of VGLUT3 expression in 6-OHDA-lesioned rats (Figures 3C, i-l and 4C, i-l). Interestingly, this effect was particularly marked in the thalamus (VL/VM) (+106% versus non-stimulated 6-OHDA-lesioned rats, *p* < 0.05, *n* = 10) and the somatosensorial cortex (+99% versus non-stimulated 6-OHDA-lesioned rats, *p* < 0.05, *n* = 10).

## Discussion

The key findings of this study were: 1) DA depletion decreased VGLUT2 (−40 to -50%) in all brain structures studied; 2) STN-HFS did not affect VGLUT1-3 expression in control (sham-operated) rats whatever the brain structure analyzed; 3) STN-HFS increased VGLUT1-3 expression in 6-OHDA-lesioned rats in almost all structures analyzed. Thereby STN-HFS: i) normalized VGLUT2 levels after the decrease induced by DA depletion, and ii) significantly increased VGLUT3 levels above those detected in control animals. This was also true for VGLUT1 levels but only for the striatum.

This remodeling suggests that the mode of action of STN-HFS results from a global effect on basal ganglia network and related structures and that its therapeutic efficacy may to be linked, at least in part, to the normalization of thalamostriatal and thalamocortical neurotransmissions.

### Bilateral effects of unilateral DA lesioning and STN-HFS on VGLUT expression

As stated above, unilateral DA lesioning and STN-HFS caused similar changes in VGLUT expression on both sides of the brain. Bilateral effects of unilateral DA lesioning have already been reported in the striatum for tissue concentrations of glutamate [9], extracellular glutamate content assessed by microdialysis or voltammetry [34,40], glutamate receptor mRNA [41] and the glial glutamate transporter GLT-1 [42]. Similarly, unilateral STN-HFS has been reported to induce bilateral increases in striatal and nigral glutamate content [14,15,34]. These bilateral effects may result from crossed glutamatergic projections from the cortex or the thalamus (VL/VM) innervating the BG on the contralateral side, consistent with cross-talk in cortico-BG-cortical loops [42].

### Effect of 6-OHDA-SNc-lesioning on VGLUT expression

We found here that 6-OHDA-lesions had no effect on VGLUT1 or VGLUT3 levels in any of the structures studied. By contrast, VGLUT2 levels decreased significantly three weeks after lesioning. At first glance, our observations contrast with previous reported data showing that dopamine depletion is associated with an increase in synaptic glutamate release [43-45] and with high striatal extracellular glutamate levels and glutamatergic activity [9,11,34,44,46-48] and greater thalamostriatal activity [48,49], two to four weeks after lesioning of nigral dopaminergic neurons. However, other studies have reported an absence of change in glutamate levels [50]. These differences may be accounted for by differences in the extent of the dopamine lesion, lesion sites, methodologies and time courses. We cannot exclude the possibility that different cellular mechanisms underlie presynaptic glutamate processes and extracellular glutamate release after lesioning. Indeed, striatal extracellular glutamate levels have been reported to depend on a complex balance between vesicular release and non vesicular release via glutamate transporters on both neurons and glia and the cysteine-glutamine antiporter [50,51]. Dopamine depletion leads to complex, biphasic changes in striatal glutamatergic transmission over the first few weeks, possibly stabilizing over three months. Contradictory data have been reported, for the cysteine-glutamate antiporter [52] and glial transporters [42,53,54] for example.

Furthermore, the changes in VGLUTs expression induced by 6-OHDA lesions are also complex. VGLUT1 levels increase in the three weeks following the injection but then decrease, whereas VGLUT2 levels decrease and then normalize [42,55-57]. In monkeys, MPTP treatment increases VGLUT1 expression but does not affect VGLUT2 levels [53,58]. In postmortem samples of Parkinsonian patients VGLUT1 and VGLUT2 levels are increased in the putamen while VGLUT1 levels is lowered in the prefrontal and temporal cortex [56].

However, the decrease in VGLUT2 levels observed here in all the brain structures of 6-OHDA rats closely parallels the thalamic hypoactivity induced by the strengthening of GABAergic inputs from the SNr and EP/GPi by the STN overactivity observed in DA-depleted BG networks [59]. Furthermore, neuronal degeneration has been observed postmortem in the thalamic nuclei of PD patients [60] and in the parafascicular nucleus in 6-OHDA-lesioned rats [49]. VGLUT2 is massively expressed by thalamic nuclei [61] and have be postulated as selective marker of thalamo-striatal activity [62]. This observation support the notion of a potential decreased glutamatergic afferences from the thalamus (VL/VM).

### Effect of STN-HFS on VGLUT1-3 expression in control and 6-OHDA-lesioned rats

STN-HFS had no effect on VGLUT1-3 expression in any of the brain structures studied in control rats. This suggests that in the absence of dopamine depletion, STN-HFS did not affect VGLUT1-3 expression. These data are rather surprising since we reported in previous microdialysis study that in intact rats STN-HFS increases extracellular glutamate in the striatum, the globus pallidus and the SNr [14-16,34]. However, the duration of stimulation used here was longer than that used in our previous studies. Thus, we can speculate that increase of extracellular glutamate levels induced by STN-HFS in physiological conditions mainly involves non vesicular release. By contrast, after 6-OHDA lesions, STN-HFS induced an increase in VGLUT1-3 expression in almost all the structures analyzed. In these DA depleted conditions, we cannot exclude the possibility that the balance between non vesicular and vesicular release of glutamate is disturbed, involving more glutamate transporters on both neurons and glia [50,51]. Indeed, it is well documented that following DA nigrostriatal lesion, there is an increase in the number of glial cells, including astrocytes and microglia. Therefore, the non vesicular release may be due to an increase in membrane transporters, such as glial glutamate transporters (GLT1 and GLAST) and the neuronal glutamate transporter EEAC1 [55,63,64]. Thus, our previous findings concerning increased extracellular glutamate levels in the striatum, globus pallidus and SNr of 6-OHDA-lesioned rats [15,16,34] are consistent and confirm that STN-HFS affects not only its direct targets, but also more distant structures of the BG network [32,33]. The mechanisms underlying the therapeutic effects of STN-HFS are not fully elucidated. STN neuron inhibition by HFS, with loss of the drive of the internal part of the globus pallidus and disinhibition of the thalamus (VL/VM), would be consistent with the classical BG model [59,65]. However, far more complex effects and circuitry are probably involved. For example, the direct activation of nearby thalamostriatal and pallidonigral fibres [16,66] or direct or antidromic cortex activation [67,68]. These mechanisms might lead to corticostriatal fiber activation and the observed increase in VGLUT1 levels.

Similarly, the increase in VGLUT2 levels induced here by STN-HFS may reflect a release of the classical thalamic inhibition induced by dopamine lesions. As VGLUT2 is found mostly in the thalamostriatal neurons specifically affected by DA depletion, these data strongly suggest a major role for this pathway in the therapeutic effects of STN-HFS in PD [69,70]. However, the normalization of VGLUT2 levels we observed in the somatosensory and premotor cortices may also play a non negligeable role in these effects of STN-HFS.

Interestingly, VGLUT2 was also overexpressed in the SNr following STN-HFS in 6-OHDA-lesioned rats. As VGLUT2 is the only VGLUT expressed by STN glutamatergic projections to the SNr [71], this confirms our hypothesis that information transmission via the transsubthalamic pathway is not completely blocked during STN-HFS [15,16,31,32,67,68] although the effect on VGLUT2 expression in the SNr might also be mediated by the modulation of thalamic afferents during STN-HFS.

VGLUT3 is expressed in striatal cholinergic interneurons linked to the dopaminergic, GABAergic and glutamatergic systems in the cortico-BG-cortical loop [38]. It is therefore unsurprising that STN-HFS, which affects the whole BG network, affects VGLUT3 expression in the striatum and related structures, such as the cortex, hippocampus, nucleus accumbens, thalamus (VL/VM) and amygdala. Indeed, STN-HFS has been reported to affect not only dopamine, glutamate and GABA [14-16,34,36,68] but also serotonin and cholinergic transmissions [72-74]. Consequently, it is difficult to explain the role of VGLUT3 in STN-HFS mechanisms. However, VGLUT3 in cholinergic interneurons has been shown to increase acetylcholine tone and to release both glutamate and acetylcholine [18]. This mechanism might explain the efficacy of STN-HFS, via complex modulation of the BG network [32,33].

## Conclusion

In conclusion, this is the first study of the expression of the three VGLUT subtypes in various brain structures in control and 6-OHDA-lesioned rats subjected to STN-HFS. We know that all the three types of VGLUTs are expected to be found in axon terminals and that immunoradioautography cannot really disclose between neuronal vs. axonal vs. glial expression of VGLUTs. In situ hybridization looking for different VGLUT transcripts will probably provide some added morphological value to our present study. These experiments are planned in our lab. However, our present data can suggest that STN-HFS may achieve its therapeutic effect, at least in part, through normalization of the thalamostriatal and the thalamocortical pathways.

## Competing interests

The authors declare that they have no competing interests.

## Authors’ contributions

Conceived and designed the experiments: MF, SB, SEM, MS. Performed the experiments: MF, GD, CC. Analyzed the data: MF, CC, MS. Contributed reagents/materials/analysis tools: SEM, MS. Wrote the paper: MF, SB, SEM, MS. All authors read and approved the final manuscript.
